# Supplementary material for: The Imaging Database for Epilepsy And Surgery (IDEAS)
Source: Epilepsia. 2024 Dec 5;66(2):471–81. doi: 10.1111/epi.18192 (PMC11827737; doi:10.1111/epi.18192)
Supplement: Supplementary file 1 — Tables S1–S3. [file EPI-66-471-s001.docx]

**Supplementary**

**Control Datasets**

We used a large number of controls as a healthy, normative baseline. These included 100 control scans acquired on the same scanner(s) as the patient cohort. In addition, we used publicly available healthy control data from the NKI (n=833) and OASIS (n=542) datasets to account for known healthy effects in cortical thickness and subcortical volume.

The age and sex distribution of the control (and patient) datasets are quantified in Table S1. The proportion of females in each control cohort was no different from patients when accounting for multiple comparisons (UCL: p=0.19; NKI: p=0.02; OASIS: p=0.02). Testing of proportional differences used a Chi-squared test with a Bonferroni-corrected threshold for significance at 0.05 / 3 ≈ 0.017.

Controls in the OASIS dataset were older than patients (p<0.001), but these were only used to model the effects of healthy aging. Controls in the UCL dataset were also marginally older than controls (p=0.005), but the effects of healthy aging were accounted for before abnormalities (z-scores) were calculated.

| **Table S1: Sex and age information for each cohort.** | | | |
| --- | --- | --- | --- |
| Cohort | Number of Subjects | Number (Proportion) of Female Subjects | Median Age at scan, years (IQR) |
| Patients (UCL) | 442 | 238 (54%) | 34 (18, 50) |
| Controls (UCL) | 100 | 62 (62%) | 39 (29, 50) |
| Controls (NKI) | 833 | 508 (61%) | 37 (18, 57) |
| Controls (OASIS) | 542 | 335 (62%) | 67 (60, 72) |

**Table S2: Sample sizes corresponding to Figure 2.**

| At Risk |  | **Years since surgery** | | | | | |
| --- | --- | --- | --- | --- | --- | --- | --- |
|  |  | **0** | **1** | **2** | **3** | **4** | **5** |
| **Resection type** | **ATLRx** | 297 | 297 | 200 | 153 | 116 | 90 |
|  | **TLesx** | 35 | 35 | 21 | 17 | 13 | 10 |
|  | **ETLesx** | 23 | 23 | 12 | 10 | 6 | 4 |
|  | **ETLx** | 54 | 54 | 29 | 19 | 16 | 11 |
|  | **Hx** | 2 | 2 | 2 | 2 | 2 | 2 |

| Event* |  | **Years since surgery** | | | | | |
| --- | --- | --- | --- | --- | --- | --- | --- |
|  |  | **0** | **1** | **2** | **3** | **4** | **5** |
| **Resection type** | **ATLRx** | 0 | 88 | 116 | 138 | 149 | 152 |
|  | **TLesx** | 0 | 9 | 10 | 11 | 13 | 14 |
|  | **ETLesx** | 0 | 11 | 11 | 11 | 12 | 12 |
|  | **ETLx** | 0 | 21 | 25 | 25 | 27 | 28 |
|  | **Hx** | 0 | 0 | 0 | 0 | 0 | 0 |
| * event = cumulative seizure recurrence since surgery | | | | | | | |

ATLRx: Anterior temporal lobe resection. TLesx: Temporal lesionectomy.

ETLesx: Extratemporal lesionectomy. ETLx: Extratemporal lobe resection.

Hx: Functional hemispherectomy.

**Table S3**

See also [www.cnnp-lab.com/ideas-data](http://www.cnnp-lab.com/ideas-data)

| Link title and URL | Description |
| --- | --- |
| Bids <https://figshare.com/s/07fca72410094bc49506> | Raw T1w and FLAIR scans organised in BIDS format. Nifti and json descriptors included |
| Masks  <https://figshare.com/s/31ab43d1829b12ac13e8> | Resection masks for IDEAS cohort in native, and freesurfer orig.mgz space |
| Freesurfer_brain  <https://figshare.com/s/39b61a1df5fa8443e3c4> | skullstripped brain from freesurfer in nifti format |
| Freesurfer_orig  <https://figshare.com/s/f13391a4161b807ce6b0> | freesurfer orig.mgz converted to nifti format |
| Freesurfer_zip  <https://figshare.com/s/b13b8bb41390d3f7a088> | freesurfer surface and volumetric reconstructions |
| Tables_stats_freesurfer  <https://figshare.com/s/010142dd51e37ba4e4e2> | Freesurfer thickness, volume, and surface areas for the Desikan-Kiliany parcellation. |
| Tables_metadata  <https://figshare.com/s/bab70268afeb1071202b> | clinical and demographic metadata |
| Table_resected  <https://figshare.com/s/097ba0e254e36f0eee52> | table indicating the percentage of each brain region in the Desikan-Kiliany atlas subsequently resected by surgery. |
| Tables_zscores  <https://figshare.com/s/8c086fc295a75f85e628> | Freesurfer thickness, volume, and surface areas for the Desikan-Kiliany parcellation, z-scored against normative controls post-combat. |
| Tables_group_effect  <https://figshare.com/s/323db205354788c4d1f0> | Group effect size differences to controls |
